# Effects of galactooligosaccharides on maternal metabolism and the gut microbiota during pregnancy

**DOI:** 10.3389/fmicb.2025.1679308

**Published:** 2026-01-06

**Authors:** Jiayang Wan, Lin An, Zhenghong Ren, Huixia Yang, Jingmei Ma

**Affiliations:** 1 Department of Obstetrics and Gynecology, Peking University First Hospital, Beijing, China; 2 Department of Gynecology, The First Affiliated Hospital, Zhejiang University School of Medicine, Hangzhou, China; 3 Department of Maternal and Child Health, School of Public Health, Peking University, Beijing, China; 4 Beijing Key Laboratory of Maternal Fetal Medicine of Gestational Diabetes Mellitus, Peking University First Hospital, Beijing, China

**Keywords:** metabolism, short chain fatty acids, gut microbiota, pregnancy, galactooligosaccharides, gestational diabetes mellitus

## Abstract

**Background:**

The gut microbiota of pregnant women changes dynamically throughout gestation, adapting to the physiological changes of pregnancy. At the same time, dysbacteriosis is involved in the pathophysiological processes of pregnancy-related diseases. Research on gut microbiota and gestational diabetes mellitus (GDM) is relatively extensive, and targeting the gut microbiota may improve maternal health. Dietary supplements such as prebiotics improve metabolic immune function in pregnant women by stimulating the growth of beneficial bacteria and promoting the production of short-chain fatty acids (SCFAs). Previous animal studies suggest that prebiotic preparations derived from galactooligosaccharides (GOS) in human milk are superior to other prebiotics.

**Objective:**

This study aims to explore the systemic effects of GOS targeting the gut microbiota on the levels of metabolism, immunity, and circulating SCFAs.

**Materials and methods:**

(1) A total of 135 pregnant women with available delivery outcomes and matched BMI were included in the analysis. From the first trimester (T1), the GOS group and the control group received GOS and fructooligosaccharide (FOS) preparations, respectively. Before the intervention (T1) and after the intervention (T2), blood samples were collected from pregnant women for LC–MS metabolomic analysis and targeted detection of short-chain fatty acids. At the same time, clinical information, metabolic indicators, and the GDM incidence rate were compared between groups, and subgroup analyses were conducted for overweight and obese participants. Statistical analyses included the t-test, the nonparametric Wilcoxon test, and the *χ*^2^ test. Correlation analysis was conducted using Fisher’s exact test and Pearson’s coefficient. (2) In order to examine the correlation between targeted gut microbiota intervention and phenotypic changes, 52 pregnant women who provided stool samples before and after the intervention (i.e., T1 and T2) were analyzed. The 16S rRNA V3–V4 variable region was sequenced on the Illumina HiSeq 2,500 platform, and QIIME was used for bioinformatics analysis. The correlations among differential flora, glycolipid metabolism, inflammatory factors, and metabolites were analyzed.

**Results:**

(1) Non-target metabolites identified several metabolites with inter-group differences: cyclamate, reserpic acid, and phenylbenzimidazole sulfonic acid were relatively higher in the GOS group. Pathway analysis indicated enrichment in butyrate, propionate, and other SCFA-related metabolic pathways, as well as in cysteine and methionine metabolism. (2) The targeted metabolites of SCFAs were further analyzed, and the effects of the intervention on SCFAs were compared. After GOS intervention, the levels of acetic acid, propionic acid, butyric acid, and hexanoic acid all increased (*p* < 0.01). Among overweight and obese pregnant women with GDM, GOS increased the levels of butyric acid and hexanoic acid (*p* < 0.05). At T2, compared with the control group, hexanoic acid levels in the GOS group increased significantly (*p* < 0.01). Correlation analysis with clinical glucose and lipid metabolism indices showed that hexanoic acid was negatively correlated with total cholesterol (TCHO) (*r* = −0.415, *p* < 0.001) and LDL (*r* = −0.347, *p* < 0.01). (3) The relative abundance of different flora was correlated with glycolipid metabolism indices and inflammatory factors. The relative abundance of *Dorea* showed a negative correlation trend with TCHO and LDL. Additionally, the relative abundance of different flora was also correlated with circulating SCFAs. The relative abundance of *Dorea and Paraprevotella* showed a positive correlation trend with hexanoic acid.

**Conclusion:**

GOS preparations containing ingredients derived from human milk may target the gut microbiota to promote the production of hexanoic acid, thereby improving lipid metabolism and inflammation, and may be beneficial for overweight and obese people with GDM.

## 1 Introduction

The gut microbiota changes throughout pregnancy and is associated with the physiological adaptations of maternal metabolism (Koren et al., 2012). Disturbances in the gut microbiota may lead to pregnancy-related complications (Wang et al., 2020), such as gestational diabetes mellitus (GDM) (Crusell et al., 2018). Moreover, microbial metabolic potentials associated with dietary fiber fermentation have been identified as related to GDM status and host glycemic traits (Sun et al., 2023). Clinical trials have shown that dietary supplement interventions during pregnancy can improve maternal health through various mechanisms, including the regulation of lipid and glucose metabolism (Babadi et al., 2019; Kijmanawat et al., 2019). In non-pregnant populations, dietary fiber has been shown to modulate the gut microbiota and improve the pathophysiological status of patients with type 2 diabetes mellitus (T2DM) (Zhao et al., 2018). Moreover, variations in maternal diet during pregnancy can alter the gut microbial community, subsequently impacting maternal metabolic and inflammatory status (Gomez-Arango et al., 2018). Therefore, this study aims to improve maternal metabolic health by developing strategies to regulate the gut microbiota.

Currently, the main dietary supplements include prebiotics, probiotics, and other supplements. They regulate the composition and function of the gut microbiota and exert effects through various mechanisms, such as improving intestinal barrier function (La Fata et al., 2018) and regulating immune function (Vulevic et al., 2015). Although prebiotics are not digested or absorbed by the host, they can promote the metabolism and proliferation of beneficial bacteria in the body and are selectively utilized by host microorganisms for health benefits (Marco et al., 2021). Common prebiotics include galactooligosaccharides (GOS), fructooligosaccharides (FOS), inulin, and some dietary fibers (Gibson et al., 2017). FOS, a plant-based dietary fiber, has been shown to improve metabolism (Miao et al., 2022; Tang et al., 2023).

In recent years, animal experiments have shown that GOS may be superior to FOS in improving immune defense and the intestinal barrier (Wu et al., 2021). GOS has also been demonstrated to improve lipid metabolism (Cheng et al., 2018). GOS shares significant structural similarities with human milk oligosaccharides and consists of 2–8 sugar units, such as galactose and glucose. These structures help protect and promote a healthy infant gut microbiota (Barnett et al., 2023; Wang et al., 2023) and support neonatal immune function. In addition, GOS and sialylated structures can regulate epithelial barrier function by inducing cell differentiation and promoting epithelial wound repair, with distinct effects on microbial composition. Specifically, they promote the growth of *Bifidobacterium* and *Bacteroides*, respectively, resulting in characteristic changes in SCFAs (Perdijk et al., 2019).

GOS prebiotics may promote SCFA production by stimulating the growth of butyrate-producing bacteria, thereby improving the health of pregnant women (Carlson et al., 2017). Most SCFAs are absorbed by colon cells and the liver as their energy source, while others are metabolized by muscle and adipose tissue (van der Beek et al., 2015). In addition, SCFAs activate GPR41 and GPR43 on intestinal epithelial cells to participate in immune-metabolic responses (Kim et al., 2013). Meanwhile, maternal circulating levels of acetate, propionate, and butyrate are correlated with demographic factors (Wang et al., 2022). For obese pregnant women, serum SCFA levels are related to some key metabolic parameters of the mother and newborn, such as propionate, which has a protective effect (Priyadarshini et al., 2014).

Recently, the beneficial effects of GOS prebiotics in clinical applications during pregnancy remain unclear. Prebiotics may regulate the gut microbiota and promote health. This study aims to compare the effects of GOS and FOS application during the first and second trimesters in pregnant women and to explore the systemic effects of GOS targeting the gut microbiota on glucose and lipid metabolism, immunity, and circulating metabolites.

## 2 Materials and methods

### 2.1 Study participants and intervention

The study was based on a registry cohort (ChiCTR1800017192), from which 150 eligible participants were enrolled according to the inclusion and exclusion criteria. Finally, 135 pregnant women with available birth outcomes and matched BMI underwent specific omics testing, and key procedures in this study were subjected to in-depth omics analysis and clinical relevance exploration. The study was approved by the Ethics Committee of Peking University First Hospital (reference number: 164), and all patients provided informed consent. The final analysis, including the target sample size for the full registry cohort, will be reported upon the completion of the entire cohort.

The inclusion criteria were as follows: age 18–40 years; residence in Beijing; ability to understand and willingness to provide informed consent; singleton pregnancy; and attendance at the first prenatal care visit between 5 and 8 weeks of gestation. The exclusion criteria were as follows: smoking, excessive alcohol consumption or drug abuse; pregnancy complicated by chronic diseases such as pre-existing diabetes, impaired glucose tolerance, impaired fasting glucose, or chronic hypertension; use of any prescribed chronic medications; and steroid use.

Eligible female participants were randomly assigned in a 1:1 ratio to either the GOS group or the control group. The randomization process, which was stratified by four BMI categories (underweight, normal weight, overweight, and obese), was conducted using the ‘H6WORLD’ platform to generate and implement the allocation sequence. A double-blind design was maintained throughout the trial for both participants and investigators. From T1 to T2, patients in the GOS group took the GOS supplement, and patients in the control group took preparations containing FOS; the dose of the supplement was 60 g/day. The GOS group mainly consisted of GOS (6 g/100 g) and sialic acid (3 g/100 g). The control group mainly included FOS (3 g/100 g). The purity of GOS and FOS in the dry matter was 90 and 93% (w/w), respectively. The dietary supplements were provided by Beijing Sanyuan Foods Co., Ltd., Beijing, China.

### 2.2 Sample collection

The samples were collected by well-trained staff in accordance with standard operating procedures. Blood samples were collected during the first trimester (T1) and the second trimester (T2). Maternal fasting blood was drawn using blood collection tubes and centrifuged at 4000 rpm for 20 min to prepare plasma. At the same time, stool samples were collected at T1 and T2 and placed in sterile tubes. The plasma and stool samples were stored at −80 °C until analysis.

### 2.3 Anthropometrics and biochemical assessment

Clinical information was collected from the medical record system for pregnant women, such as maternal age, pre-pregnancy weight, pre-pregnancy BMI (*p*-BMI), blood pressure, last menstrual period, and weight gain. The incidence of GDM by the second trimester was calculated from the results of the 75-g oral glucose tolerance test (OGTT) performed at 24–28 weeks of gestation (Metzger et al., 2010).

Several biochemical indicators were also collected and measured at our hospital, such as white blood cell count (WBC), neutrophil count (NEU), blood glucose levels, blood lipid levels (including triglyceride (TG), total cholesterol (TCHO), high-density lipoprotein (HDL), and low-density lipoprotein (LDL)), and interleukin-6 (IL-6).

### 2.4 Metabolome analysis of LC–MS

For quality control (QC) sample preparation, we combined equal volumes of the prepared samples into a single large sample, then divided it into 17 QC samples to monitor the instrument’s precision and stability. Before sampling, 3 QC samples were used to assess instrument precision, and 1 QC sample was collected every 10 samples to assess instrument stability. The stability of the analysis was confirmed by the high degree of overlap in the total ion chromatograms (TICs) of all QC samples (Supplementary Figure S1).

Metabolites in plasma were measured. Briefly, to extract metabolites from plasma samples, 400 μL of cold extraction solvent (methanol/acetonitrile/H2O, 2:2:1, v/v/v) was added to 100 μL of the sample, and the mixture was vortexed. After vortexing, the samples were incubated on ice for 20 min and then centrifuged at 14,000 g for 20 min at 4 °C. The supernatant was collected and dried in a vacuum centrifuge at 4 °C. For LC–MS analysis, the samples were redissolved in 100 μL of acetonitrile/water (1:1, v/v) and transferred to LC vials.

Analyses were performed using ultra-high performance liquid chromatography (UHPLC) Ultimate 3,000 coupled with high-resolution mass spectrometry Q Active HF-X (Thermo Fisher Scientific, USA) using a chromatographic column (HSS T3 100 * 2.1 mm, 1.8 μm, Waters). The mobile phase in positive-ion mode consisted of water containing 0.1% formic acid (solution A) and 100% methanol containing 0.1% formic acid (solution B). In negative-ion mode, the mobile phase comprised water containing 0.05% acetic acid (solution C) and methanol containing 0.05% acetic acid (solution D). The chromatographic gradient is detailed in Supplementary Table S1. The flow rate was 0.3 mL/min, the column temperature was maintained at 40 °C, and the injection volume was 3 μL. The mass spectrometry parameter conditions are shown in Supplementary Table S2.

For data acquisition and processing, the raw MS data were converted to a universal (abf) format using the AnalysisBase File Converter software before being imported into the available MSDIAL software. During the metabolite screening period, the *p*-values obtained from the univariate statistical test (t-test) for comparing metabolite levels between groups were corrected using the Benjamini-Hochberg procedure.

### 2.5 Measurement of SCFAs by GC–MS

The content of SCFAs in plasma was determined qualitatively and quantitatively. Gas chromatography–mass spectrometry (GC/MS; Agilent 7890A GC-FID) was used to characterize the SCFAs in plasma. The GC was fitted with a capillary column (Agilent DB-FFAP, 30 m × 0.25 mm × 0.25 μm), and helium was used as the carrier gas at 5 mL/min. Injection was performed in split mode at 5:1, with an injection volume of 1 μL and an injector temperature of 250 °C (Zhang et al., 2019). Samples were thawed at 4 °C and vortex-mixed thoroughly. Approximately 0.1 g of sodium chloride was weighed into a 1.5 mL centrifuge tube, followed by the addition of 200 μL of the sample, 8 μL of internal standard, and 20 μL of 50% concentrated sulfuric acid. After vortexing for 10 s, 200 μL of anhydrous ether was added, and the mixture was vortexed for 1 min. Subsequently, the mixture was centrifuged at 13,000 rpm for 5 min, and 80 μL of the supernatant was collected for analysis. All data were processed using FID Chem Station (G1701EA.02.00.493) and ACD/Spectrum Processor 2015 (S30S41) software.

### 2.6 DNA extraction and 16S rRNA gene sequencing

To assess the correlation between targeted intervention on gut microbiota and phenotype, fecal samples collected from 52 pregnant women before and after the intervention (i.e., T1 and T2) were analyzed. Fecal DNA was extracted using a commercial kit (Qiagen, Hilden, Germany). PCR amplification of fecal DNA was performed using 16S amplicon PCR forward primers and 16S amplicon PCR reverse primers. After PCR amplification, the amplicons in each library were purified using Qiagen to prepare the library. Subsequently, the qualified library was sequenced using the Illumina HiSeq 2,500 high-throughput sequencing platform. The UCLUST algorithm was used to compare operational taxonomic units (OTUs) at 97% identity, and the Greengenes 16S rRNA database was used for taxonomic classification of 16S rRNA gene sequences. The paired read segments were merged with Flash software, with a maximum mismatch rate of 10% and an overlap length of at least 10 bases. Alpha and beta diversities were generated in Quantitative Insights Into Microbial Ecology (QIIME).

### 2.7 Statistical analysis

Data were presented as mean ± standard deviation (SD) or count (*n* (%)). Statistical analyses were carried out using SPSS (version 25.0). GraphPad Prism (version 8.0) was used to draw diagrams. The chi-squared test and Fisher’s exact test were applied for categorical variables, and the t-test or the non-parametric Wilcoxon test was used for continuous variables where appropriate. Pearson’s coefficient was used for correlation analysis. A *p*-value of < 0.05 was considered to be statistically significant.

## 3 Results

### 3.1 Participants and clinical characteristics

A total of 150 pregnant women who met the inclusion and exclusion criteria were randomly assigned to two groups, with three cases assigned to the GOS group and 12 to the control group. A total of 135 pregnant women were included in the study analysis, including 72 in the GOS group and 63 in the control group. The flow of participants and analyses through the study is shown in Supplementary Figure S2. The clinical baseline T1 values before maternal intervention are summarized in Table 1. There was no significant difference in clinical characteristics, including age, pre-pregnancy weight, *p*-BMI, and blood pressure before intervention (*p* > 0.05), and the two groups were comparable.

**Table 1:** Baseline clinical characteristics of study participants.

	GOS (*n* = 72)	Control (*n* = 63)	*p*-value
Age (years)	33.43 ± 3.22	33.79 ± 3.67	0.542
Pre-pregnancy weight (kg)	61.50 ± 10.87	61.66 ± 11.15	0.932
*p*-BMI (kg/m^2^)	22.67 ± 3.46	23.33 ± 4.03	0.304
BMI classification [*n* (%)]			0.919
Underweight (BMI<18.5 kg/m^2^)	4 (5.6)	2 (3.2)	
Normal weight (BMI 18.5–23.9 kg/m^2^)	50 (69.4)	43 (68.3)	
Overweight (BMI 24–27.9 kg/m^2^)	11 (15.3)	11 (17.5)	
Obesity (BMI > 28 kg/m^2^)	7 (9.7)	7 (11.1)	
SBP (mmHg)	114.56 ± 10.08	116.00 ± 12.02	0.449
DBP (mmHg)	66.75 ± 8.69	68.84 ± 9.79	0.191

Data presented are mean ± SD or *n* (%).

*p*-values for comparisons between the two groups in *t*-tests for continuous variables, and *χ*^2^ and Fisher’s exact tests for categorical variables.

*p*-BMI, pre- pregnancy body mass index; SBP, systolic blood pressure; DBP, diastolic blood pressure.

### 3.2 Changes in body weight and BMI from T1 to T2

As gestational age progressed, the weight gain of two groups of pregnant women from T1 to T2 was recorded, and changes in BMI were calculated (Table 2). There was no significant difference in gestational weight gain or BMI increase between the GOS and control groups (*p* > 0.05).

**Table 2:** Changes in body weight and BMI.

	GOS (*n* = 72)	Control (*n* = 62)	*p*-value
BW_T1 (kg)	61.90 ± 10.57	62.11 ± 10.55	0.906
BW_T2 (kg)	67.69 ± 10.82	67.46 ± 10.47	0.900
BW_T2-T1 (kg)	6.00 ± 2.96	5.34 ± 2.52	0.176
BMI_T1 (kg/m^2^)	22.83 ± 3.37	23.54 ± 3.93	0.257
BMI_T2 (kg/m^2^)	25.04 ± 3.51	25.56 ± 3.84	0.406
BMI_T2-T1 (kg/m^2^)	2.21 ± 1.17	2.02 ± 0.94	0.315

BW, body weight; BMI, body mass index.

### 3.3 Changes in glucose, lipid metabolism, and inflammatory indicators

The study analyzed changes in glucose and lipid metabolism, as well as inflammatory markers, in pregnant women from T1 to T2, both before and after the intervention (Tables 3, 4). There was a significant difference in HDL levels between the two groups at T2 after the intervention (1.73 ± 0.33 mmol/L vs. 1.61 ± 0.32 mmol/L; *p* < 0.05), with HDL levels increasing after the GOS intervention.

**Table 3:** Glucose, lipid metabolism, and inflammatory indicators before the intervention.

	GOS (*n* = 67, T1)	Control (*n* = 63, T1)	*p*-value
Sampling gestational week (weeks)	12.80 ± 1.71	12.87 ± 1.45	0.804
Glucose-T1 (mmol/L)	5.04 ± 0.36	5.02 ± 0.38	0.708
TG-T1 (mmol/L)	1.04 ± 0.50	1.04 ± 0.45	0.986
TCHO-T1 (mmol/L)	4.23 ± 0.82	4.21 ± 0.83	0.906
HDL-T1 (mmol/L)	1.42 ± 0.27	1.42 ± 0.26	0.940
LDL-T1 (mmol/L)	2.24 ± 0.66	2.26 ± 0.62	0.857
WBC-T1 (10^9^/L)	8.70 ± 2.19	8.31 ± 2.13	0.303
NEU-T1 (10^9^/L)	6.25 ± 1.91	5.84 ± 1.82	0.214

**Table 4:** Glucose, lipid metabolism, and inflammatory markers following intervention.

	GOS (*n* = 72, T2)	Control (*n* = 62, T2)	*p*-value
Sampling gestational week (weeks)	25.36 ± 2.27	25.60 ± 2.22	0.542
GLU 0 h (mmol/L)	4.76 ± 0.45	4.86 ± 0.57	0.268
GLU 1 h (mmol/L)	8.27 ± 1.91	8.41 ± 2.11	0.673
GLU 2 h (mmol/L)	6.97 ± 1.68	6.99 ± 1.52	0.943
TG-T2 (mmol/L)	2.23 ± 0.86	2.20 ± 0.85	0.822
TCHO-T2 (mmol/L)	5.48 ± 1.07	5.27 ± 1.11	0.251
HDL-T2 (mmol/L)	1.73 ± 0.33	1.61 ± 0.32	0.045*
LDL-T2 (mmol/L)	2.87 ± 0.78	2.80 ± 0.82	0.659
WBC-T2 (10^9^/L)	9.75 ± 2.40	9.54 ± 2.10	0.592
NEU-T2 (10^9^/L)	7.34 ± 2.09	7.16 ± 1.78	0.594

GLU, glucose; TG, triglyceride; TCHO, total cholesterol; HDL, high-density lipoprotein; LDL, low-density lipoprotein; WBC, white blood cell count; NEU, neutrophil count.

### 3.4 Diagnosis of GDM and changes in biochemical indicators in the two groups

During 24–28 weeks of pregnancy, fasting blood glucose (GLU 0 h), 1-h (GLU 1 h), and 2-h (GLU 2 h) blood glucose levels were measured using an oral glucose tolerance test (OGTT) to determine whether GDM was diagnosed. The results showed that 26 pregnant women in the GOS group were diagnosed with GDM, while 22 pregnant women in the control group were diagnosed with GDM (Table 5).

**Table 5:** GDM diagnosis and OGTT values of the GOS group and the control group.

	GOS (*n* = 72)	Control (*n* = 62)	*p*-value
Plasma glucose in OGTT (mmol/L)			
Fasting	4.76 ± 0.45	4.86 ± 0.57	0.268
1 h	8.27 ± 1.91	8.41 ± 2.11	0.673
2 h	6.97 ± 1.68	6.99 ± 1.52	0.943
GDM diagnosis [*n* (%)]			0.940
Yes	26 (36.1)	22 (35.5)	
No	46 (63.9)	40 (64.5)	

GDM, gestational diabetes mellitus; OGTT, oral glucose tolerance test.

The glucose, lipid metabolism, and inflammatory indicators (WBC, NEU) of the two groups diagnosed with GDM during T2 are listed in Table 6. The results showed that, although there was no statistically significant difference (*p* > 0.05) in glucose, lipid metabolism, and inflammation indicators between the two groups, the GOS group showed a trend of improvement compared to the control group. For example, there was a slight decreasing trend in GLU 0 h (5.07 ± 0.57 mmol/L vs. 5.35 ± 0.66 mmol/L) and GLU 1 h (9.89 ± 1.67 mmol/L vs. 10.28 ± 1.74 mmol/L) levels.

**Table 6:** Clinical and biochemical status of two groups diagnosed with GDM.

	GDM_GOS (*n* = 26)	GDM_Control (*n* = 22)	*p*-value
Age (years)	34.69 ± 3.27	34.64 ± 3.96	0.957
Sampling gestational week (weeks)	25.20 ± 2.52	25.29 ± 2.30	0.900
*p*-BMI (kg/m^2^)	23.03 ± 4.03	25.07 ± 3.82	0.080
GLU 0 h (mmol/L)	5.07 ± 0.57	5.35 ± 0.66	0.116
GLU 1 h (mmol/L)	9.89 ± 1.67	10.28 ± 1.74	0.434
GLU 2 h (mmol/L)	8.37 ± 1.55	8.31 ± 1.54	0.889
TG-T2 (mmol/L)	2.18 ± 0.94	2.35 ± 1.11	0.551
TCHO-T2 (mmol/L)	5.36 ± 1.11	5.11 ± 1.19	0.441
HDL-T2 (mmol/L)	1.72 ± 0.35	1.55 ± 0.33	0.092
LDL-T2 (mmol/L)	2.78 ± 0.75	2.66 ± 0.88	0.627
WBC-T2 (10^9^/L)	9.70 ± 1.95	9.75 ± 1.61	0.931
NEU-T2 (10^9^/L)	7.32 ± 1.70	7.37 ± 1.45	0.915

### 3.5 GDM diagnosis and biochemical indicators in overweight and obese pregnant women

Further analysis was conducted on overweight and obese pregnant women in the GOS group and control group, with the aim of exploring the effects of intervention on specific populations. Under comparable baseline conditions, for overweight and obese pregnant women, the incidence of GDM in the GOS group showed a certain decreasing trend compared to the control group (38.9% vs. 66.7%; *p* = 0.095) (Table 7).

**Table 7:** Clinical and GDM diagnosis of overweight and obese pregnant women in two groups.

	OW-OB_GOS (*n* = 18)	OW-OB_Control (*n* = 18)	*p*-value
Age (years)	33.94 ± 2.82	35.06 ± 4.18	0.356
Sampling gestational week (weeks)	24.94 ± 2.90	25.46 ± 2.42	0.566
*p*-BMI (kg/m^2^)	27.35 ± 3.00	28.01 ± 2.95	0.507
GDM diagnosis [*n* (%)]			0.095
Yes	7 (38.9)	12 (66.7)	
No	11 (61.1)	6 (33.3)	

OW-OB, overweight and obese.

The study analyzed the biochemical indicators of pregnant women diagnosed with GDM in two groups: overweight and obese individuals. Among overweight and obese individuals diagnosed with GDM, GOS intervention showed a decreasing trend in inflammatory markers, such as IL-6 (0.98 ± 0.39 vs. 1.24 ± 0.29; *p* = 0.111) (Table 8).

**Table 8:** Clinical and biochemical markers in overweight and obese pregnant women diagnosed with GDM.

	OW-OB _GDM_GOS (*n* = 7)	OW-OB _GDM_Control (*n* = 12)	*p*-value
Age (years)	35.71 ± 3.59	35.25 ± 3.98	0.803
Sampling gestational week (weeks)	23.84 ± 4.19	24.86 ± 2.23	0.494
*p*-BMI (kg/m^2^)	27.89 ± 4.12	27.90 ± 2.46	0.996
GLU 0 h (mmol/L)	5.41 ± 0.85	5.60 ± 0.71	0.592
GLU 1 h (mmol/L)	10.23 ± 2.59	9.88 ± 1.97	0.744
GLU 2 h (mmol/L)	8.26 ± 1.63	8.36 ± 1.60	0.899
TG-T2 (mmol/L)	2.30 ± 1.28	2.37 ± 1.16	0.905
TCHO-T2 (mmol/L)	5.46 ± 1.17	4.90 ± 1.22	0.344
HDL-T2 (mmol/L)	1.75 ± 0.51	1.47 ± 0.34	0.174
LDL-T2 (mmol/L)	2.75 ± 0.73	2.53 ± 0.84	0.56
WBC-T2 (10^9^/L)	9.89 ± 2.05	10.15 ± 1.73	0.772
NEU-T2 (10^9^/L)	7.49 ± 1.87	7.59 ± 1.65	0.904
IL-6-T2 (pg/mL)	0.98 ± 0.39	1.24 ± 0.29	0.111

IL-6, interleukin-6.

### 3.6 Differential metabolite analysis

**Figure 1:**
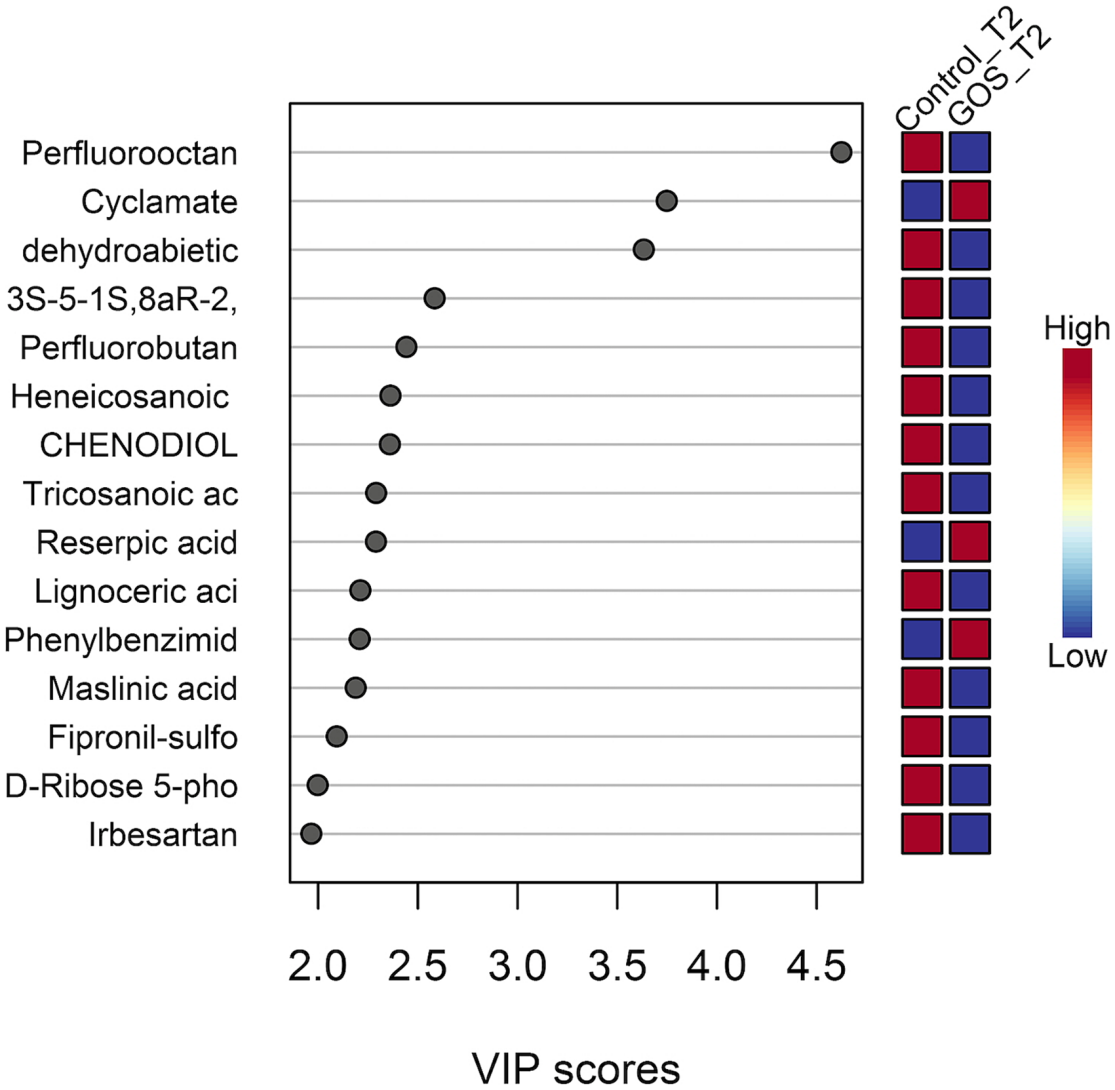
Differential metabolite analysis based on PLS-DA. Variables with VIP values greater than 1 show significant differences; the higher the VIP value, the greater its contribution to sample differentiation. The color coding on the right indicates metabolite concentrations across different groups.

To identify differential metabolites between the two groups, a Partial Least Squares-Discriminant Analysis (PLS-DA)-based Variable Importance in Projection (VIP) plot was used to assess variable importance and their contributions to sample differentiation (Figure 1). The top 15 differential metabolites listed in the figure include perfluorooctanoic acid, cyclamate, dehydroabietic acid, and others. In the GOS group, the relative concentrations of cyclamate, reserpic acid, and phenylbenzimidazole sulfonic acid were relatively high.

### 3.7 Metabolic pathway analysis

**Figure 2:**
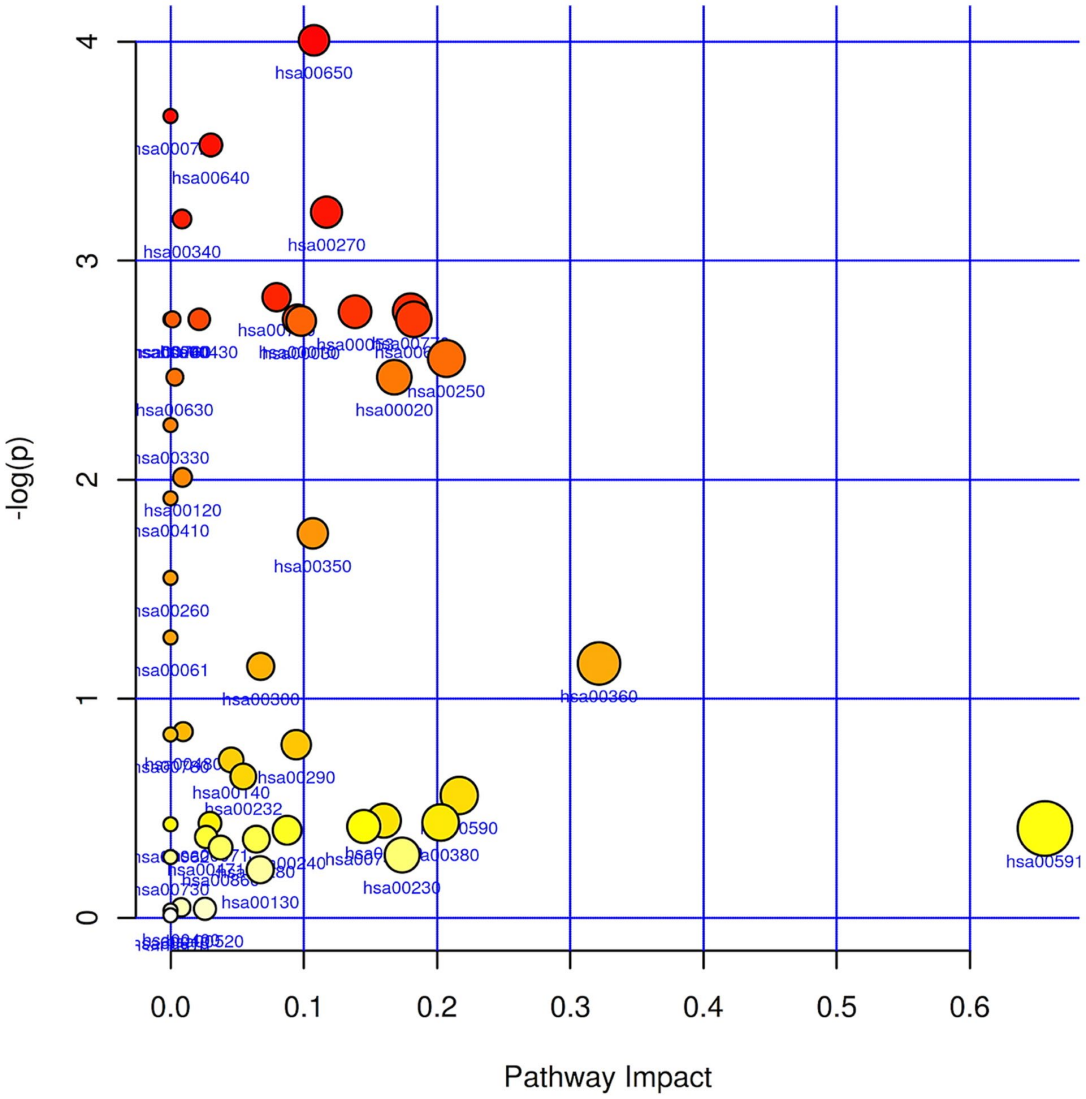
Metabolic pathway analysis. The pathway impact value along the horizontal coordinate represents the influence of the metabolic pathway, and the larger the -log(*p*) value in the vertical coordinate, the smaller the *p* value and the higher the significance. The size and color of the circle are positively correlated with these two indices above.

Pathway analysis of the two groups of samples revealed 49 metabolic pathways (Figure 2). Specific results for some relevant metabolic pathways identified are also listed (Supplementary Table S3). Differential metabolic pathways with smaller *p*-values and larger Pathway-Impact values were selected. Possible metabolic pathways observed in plasma samples included butanoate metabolism, propanoate metabolism, and cysteine and methionine metabolism (*p* < 0.05).

Butyrate in metabolic pathways identified three metabolites: (R)-3-hydroxybutyric acid, pyruvic acid, and succinic acid. Two distinct metabolites, 2-ketobutyric acid and succinic acid, were identified in the propionate metabolic pathway. The cysteine and methionine metabolic pathways identified three metabolites: 2-ketobutyric acid, pyruvate, and S-adenosylhomocysteine. Furthermore, we observed that in the enriched butanoate metabolism and propanoate metabolism pathways, most of their intermediates were detected as increased in the GOS group, suggesting that these pathways might be upregulated in the GOS group.

### 3.8 The content levels and changes of circulating SCFAs

**Figure 3:**
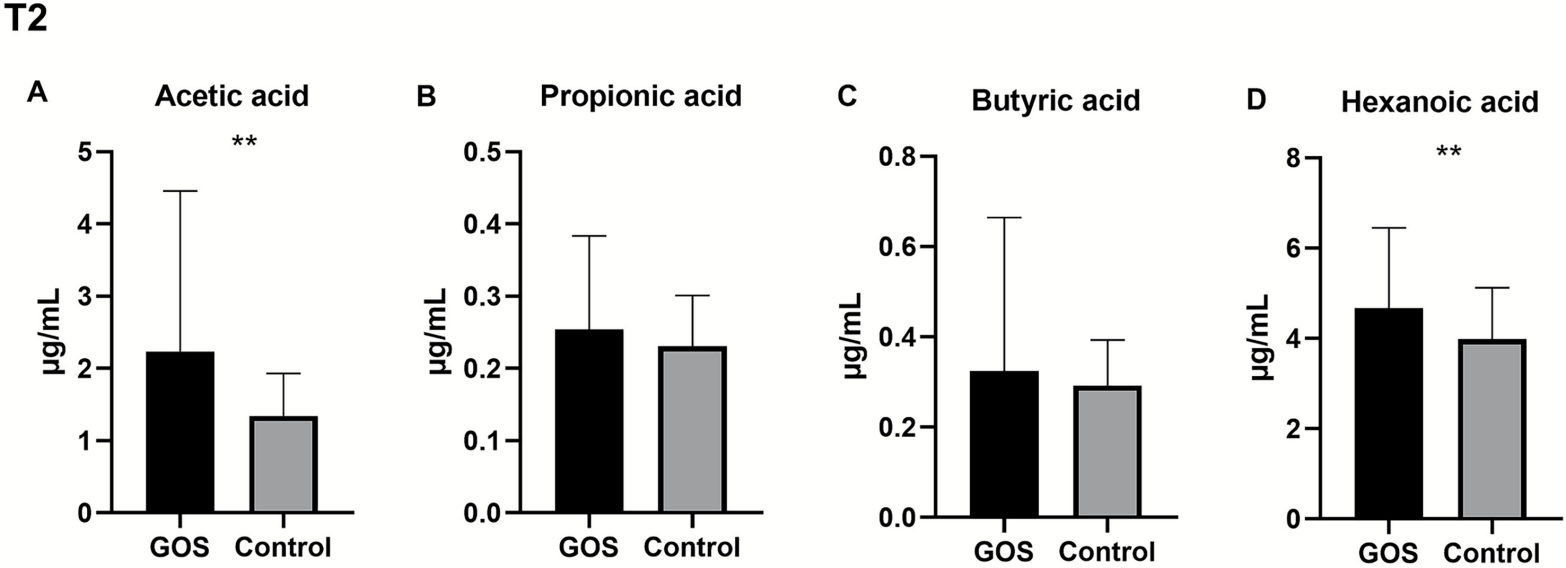
SCFA content levels in T2. **(A–D)** The circulating levels of acetic acid, propionic acid, butyric acid, and hexanoic acid in the GOS and control groups in T2.

The levels of circulating SCFAs were measured during pregnancy following the intervention. Four types of SCFAs were detected in plasma samples during pregnancy: acetic acid, propionic acid, butyric acid, and hexanoic acid. The content of SCFAs in T1 samples was tested (Supplementary Figure S3), and the content in T2 samples increased compared to T1 samples. The T2 levels of SCFAs in the GOS and control groups differed. Compared with the control group, the levels of acetic acid and hexanoic acid in the GOS group were significantly higher (*p* < 0.01). The levels of propionic acid and butyric acid in the GOS group showed a non-significant increase (*p* > 0.05). The levels of SCFAs in the two groups during mid-pregnancy are shown in Figure 3.

**Figure 4:**
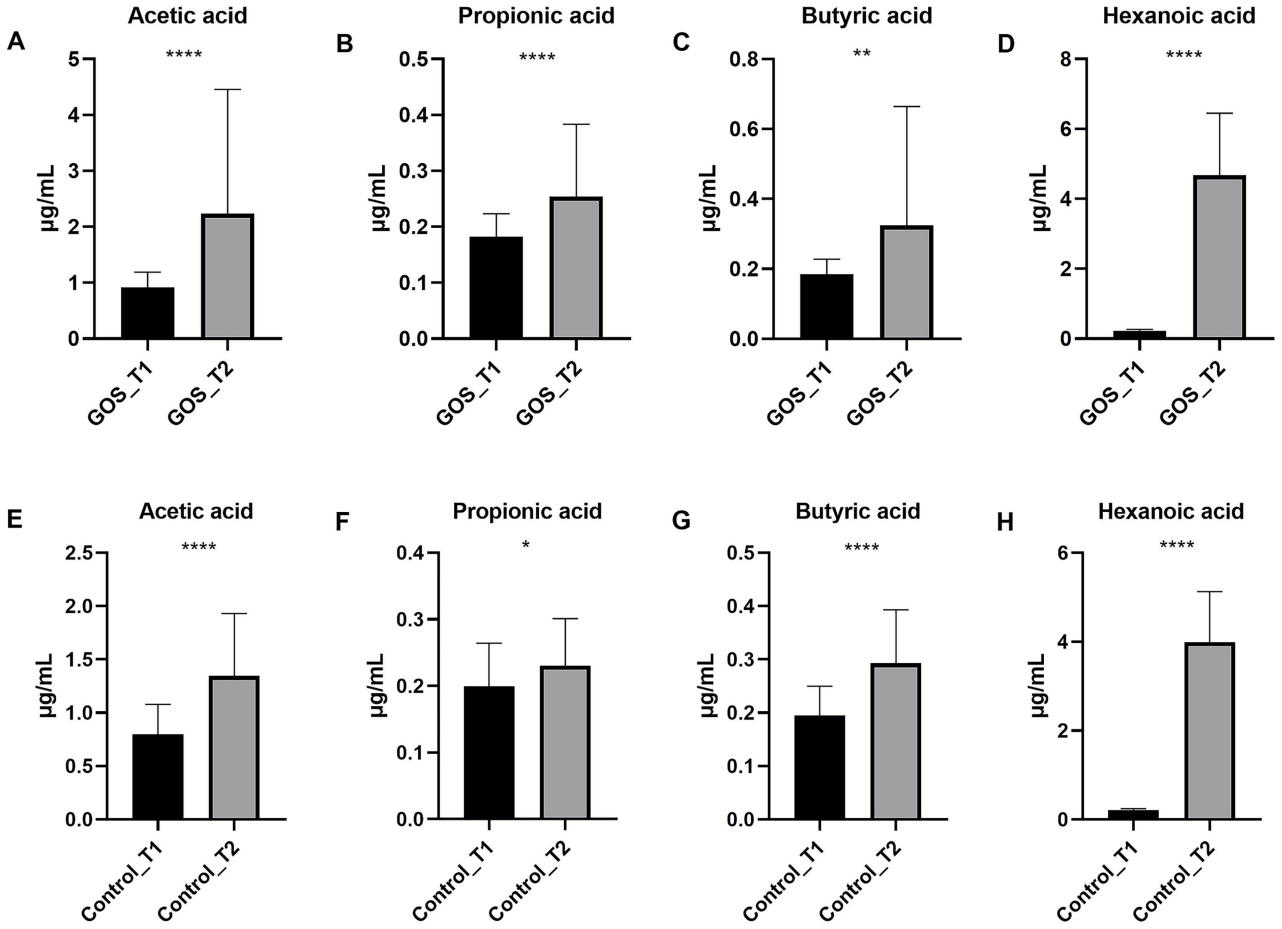
Changes in SCFA content levels in two groups before and after intervention. **(A–D)** The circulating levels of acetic acid, propionic acid, butyric acid, and hexanoic acid in the GOS group at T1 and T2. **(E–H)** The circulating levels of acetic acid, propionic acid, butyric acid, and hexanoic acid in the control group at T1 and T2.

We continued to analyze the changes in maternal circulation SCFAs before and after intervention (T1 to T2) in both groups (Figure 4). The results showed that circulating SCFA levels increased after GOS intervention, with significant increases in acetic acid, propionic acid, butyric acid, and hexanoic acid (*p* < 0.01), indicating that GOS intervention can increase circulating SCFA levels.

To investigate changes in circulating SCFA levels after intervention in specific populations, the following analysis was conducted (Supplementary Figure S4). Among overweight and obese pregnant women diagnosed with GDM, the levels of SCFAs in the GOS and control groups were numerically increased after the intervention. Compared with before the intervention, the levels of acetic acid, butyric acid, and hexanoic acid in the control group increased significantly (*p* < 0.01), and the levels of butyric acid and hexanoic acid in the GOS group increased significantly (*p* < 0.05).

### 3.9 Correlation analysis of circulating SCFAs and lipid metabolism

**Figure 5:**
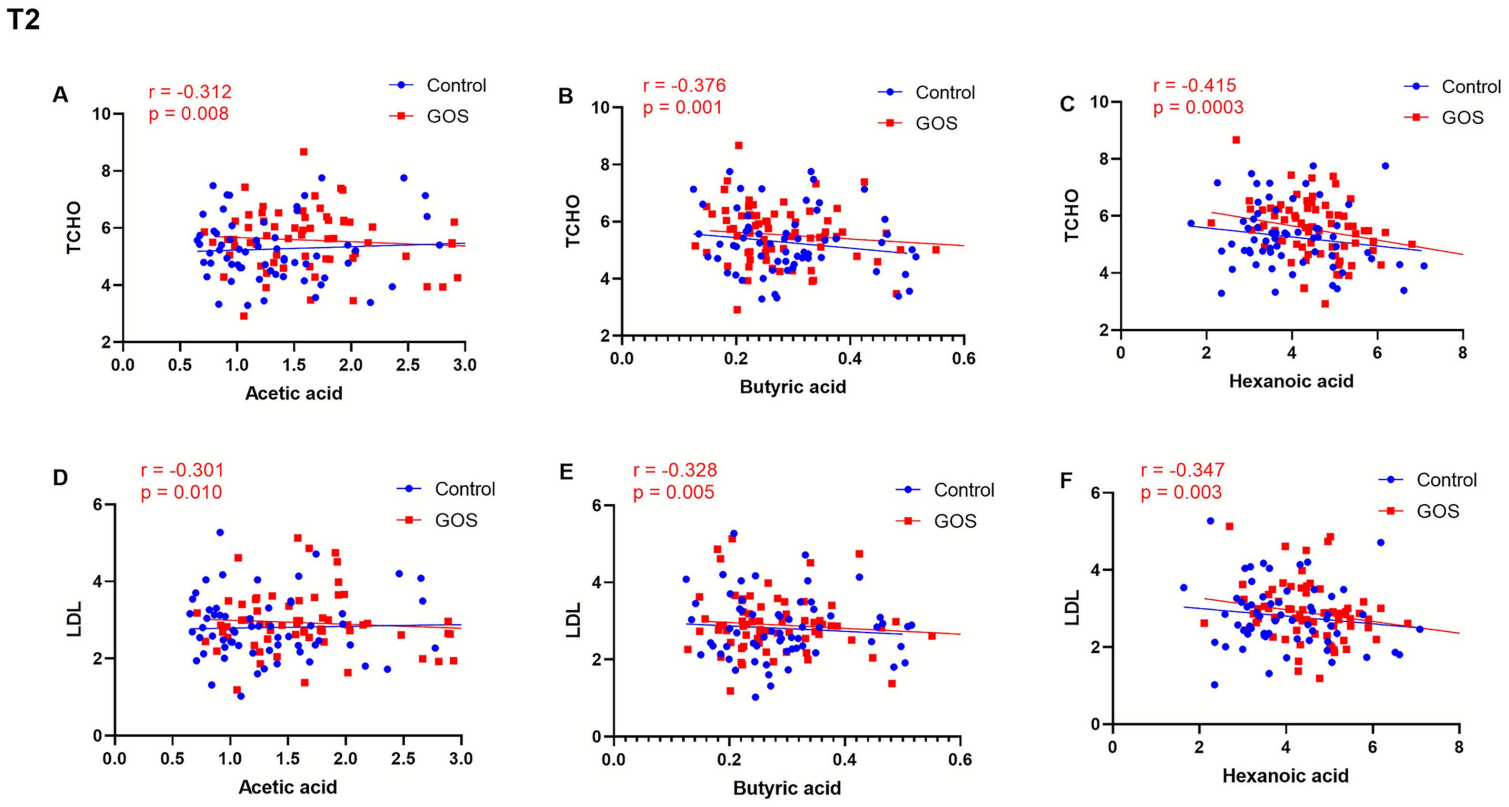
Correlation analysis of acetic acid, butyric acid, and hexanoic acid content levels and lipid metabolism indexes in T2. **(A,D)** The associations between acetic acid and TCHO or LDL in either group. **(B,E)** The associations between butyric acid and TCHO or LDL in either group. **(C,F)** The associations between hexanoic acid and TCHO or LDL in either group.

In T2, acetic acid, butyric acid, and hexanoic acid were associated with some lipid metabolism-related indicators (Figure 5). Acetic acid was negatively correlated with TCHO (*r* = −0.312, *p* = 0.008) and LDL (*r* = −0.301, *p* = 0.010) (Figures 5A,D). Butyric acid showed a negative correlation with both TCHO (*r* = −0.376, *p* = 0.001) and LDL (*r* = −0.328, *p* = 0.005) (Figures 5B,E). Hexanoic acid was also associated with the two lipid metabolism indicators mentioned above, specifically negatively correlated with TCHO (*r* = −0.415, *p* = 0.0003) and LDL (*r* = −0.347, *p* = 0.003) (Figures 5C,F). In T2, compared with the control group, the lipid metabolism indicators in the GOS intervention group showed a stronger correlation with SCFA content.

### 3.10 Association of glycolipid metabolism, inflammatory factors, and circulating SCFAs with microbiome

**Figure 6:**
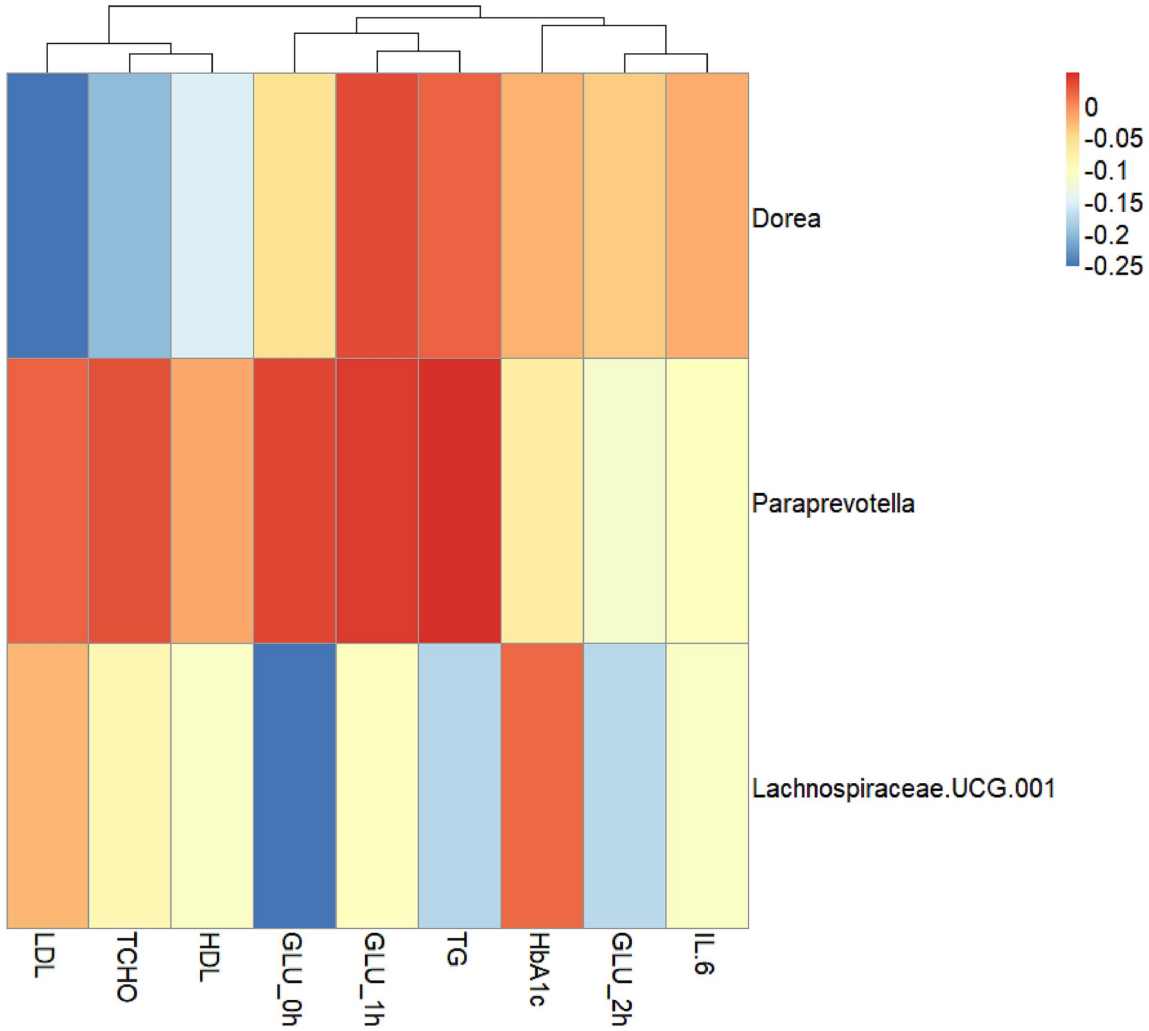
The correlation between the abundance of differential flora and glycolipid metabolism, and inflammatory factors in T2. The correlation heatmap between the abundance of differential flora and LDL, TCHO, HDL, TG, GLU 0 h, GLU 1 h, GLU 2 h, HbA1c, and IL-6. Red squares and blue squares indicate positive and negative associations, respectively.

The overall microbial structure and changes in alpha and beta diversity of the gut microbiota in the GOS and control groups have been described in detail in previous studies (Wan et al., 2023). In order to explore the relationship between changes in gut microbiota, glucose and lipid metabolism indicators, and levels of inflammatory factors, the abundance of differential flora in T2 was analyzed for correlation with glucose metabolism indicators (GLU 0 h, GLU 1 h, GLU 2 h, and HbA1c), lipid metabolism indicators (TG, TCHO, HDL, and LDL), and inflammatory factor IL-6 (Figure 6). The heat map showed no significant differences among the three bacteria and the various indicators. The relative abundance of *Dorea* showed a negative correlation with TCHO and LDL. Further correlation analysis was performed between the abundance of bacteria at the genus level after intervention and the indices of glycolipid metabolism and inflammatory factors (Supplementary Figure S5). The correlation analysis of the above biochemical indicators and omics data suggested that the relative abundance and functions of different bacterial species may affect the levels of glucose and lipid metabolism indicators and inflammatory factors at the genus level.

**Figure 7:**
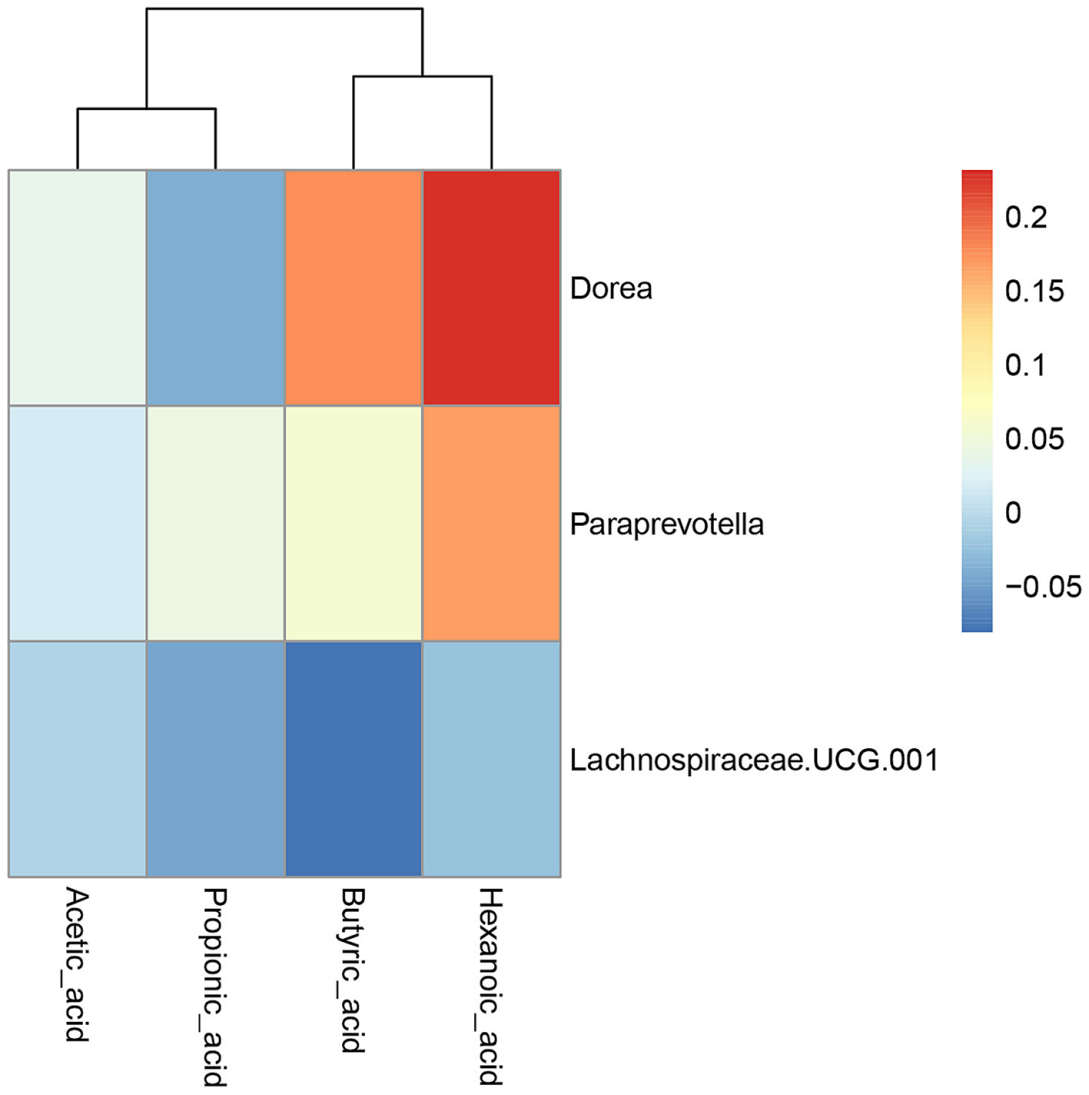
The correlation between the abundance of differential flora and SCFAs in T2. The correlation heatmap between the abundance of differential flora and acetic acid, propionic acid, butyric acid, and hexanoic acid. Red squares and blue squares indicate positive and negative associations, respectively.

To investigate the relationship between changes in gut microbiota and circulating SCFAs, a correlation analysis was conducted between the abundance of differential flora in T2 and SCFAs (acetic acid, propionic acid, butyric acid, and hexanoic acid) (Figure 7). The heat map showed no significant difference among the three bacteria and the SCFAs. The relative abundance of *Dorea* and *Paraprevotella* showed a certain positive correlation with hexanoic acid, while the relative abundance of *Dorea* showed a certain positive correlation with butyric acid. Further correlation analysis was performed between bacterial abundance at the genus level in T2 after intervention and circulating SCFAs (Supplementary Figure S6). The above metabolomic and microbiomic association analyses suggested that GOS prebiotics with breast milk-derived components may promote SCFA production by targeting the gut microbiota, thereby improving lipid metabolism and inflammation and benefiting pregnant women’s health.

## 4 Discussion

In this study, a comparative analysis was conducted between the GOS group and the control group diagnosed with GDM. Although no statistically significant differences were observed in glucose, lipid metabolism, or inflammation indicators between the two groups, the GOS group showed a slight downward trend in fasting blood glucose and 1-h blood glucose compared with the control group. A clinical study also found that taking probiotic supplements for 6 weeks in GDM patients was beneficial for blood sugar control, TG, and VLDL levels (Karamali et al., 2016). Further analysis of overweight and obese pregnant women in the GOS group and control group showed that compared to the control group, the incidence of GDM in the GOS group showed a certain downward trend. Weight and other high-risk pregnancy conditions may be the factors that affect dietary supplementation and the effectiveness of GDM prevention. Dietary interventions containing prebiotics may promote weight loss in obese patients by regulating specific gut microbiota. Compared with the placebo group, the prebiotic group showed specific changes in gut microbiota composition, increasing levels of *Bifidobacterium* (*B. bifidum*, *B. longum subs. Longum*, *B. adolescentis*) (Hiel et al., 2020). Having GDM may affect the sensitivity of pregnant women’s gut microbiota, limiting their ability to respond to dietary regulation. Overweight and obese pregnant women without GDM may benefit from a diet targeting the microbiota (Mokkala et al., 2021). The clinical application of dietary supplements in high-risk pregnant women with overweight and obesity needs further exploration.

This study continued to examine the effects of prebiotic interventions on some metabolites, using LC–MS for metabolite analysis. Based on the VIP values of the PLS-DA model, we identified differential metabolites between the GOS and control groups. In the GOS group, cyclamate, reserpinic acid, and phenylbenzimidazole sulfonic acid were relatively high, which may be metabolites that distinguish the two and contribute to sample differentiation. Based on the metabolic changes in the two groups of samples, pathway analysis was carried out, which may involve butyrate, propionate, cysteine, and methionine metabolism. Among them, butyrate was the main energy source for intestinal epithelial cells and the main substrate for metabolic reactions (Bamberger et al., 2018). It promoted the growth of intestinal epithelial cells, enhanced epithelial barrier function, and prevented “intestinal leakage” and GDM (Hasain et al., 2020). Butyrate may be closely related to most clinical information of pregnancy and has an anti-diabetic effect. Based on preliminary non-targeted metabolomics results, targeted detection of SCFA-related metabolites was conducted.

Given that approximately 95% of colon SCFAs are absorbed into the bloodstream and are related to metabolic health, the concentration of SCFAs in maternal circulation was measured (Müller et al., 2019). Our study observed an increase in SCFA levels after intervention, with significant increases in acetic acid and hexanoic acid levels in the GOS group compared to the control group. Usually, prebiotic supplements may have beneficial effects on health by stimulating beneficial bacteria and SCFAs, such as improving the intestinal environment, and the produced SCFAs may reduce lumen pH while preventing the growth of some pathogens (Ríos-Covián et al., 2016). Meanwhile, SCFAs may activate GPR41/43 in intestinal epithelial cells, promoting protective immune production (Kim et al., 2013). In addition, prebiotic intake selectively modulated *Bifidobacteria* and decreased SCFA concentrations in obese women (Salazar et al., 2014). Furthermore, the association between SCFAs and some clinical indicators was determined. Some SCFAs, such as acetic acid, butyric acid, and hexanoic acid, were negatively correlated with TCHO and LDL lipid metabolism indicators, respectively, which may be related to feedback regulation during metabolic imbalance. Different SCFAs may play distinct roles; for example, acetic acid and propionic acid act in opposite ways in adipogenesis and liver cholesterol production (Weitkunat et al., 2016). Furthermore, future research should better include concurrent measurement of fecal and serum SCFAs to fully reveal the causal pathway from dietary intervention to bacterial activity to systemic host effects.

Dietary supplements, such as prebiotics, may improve maternal health by stimulating the growth of beneficial bacteria and promoting the production of SCFAs. Moreover, GOS regulates gut microbiota, plasma immunoglobulins, and offspring gut microbiota, promoting growth and development (Wu et al., 2021). Specific microbiota can also serve as targets for early diagnosis and treatment interventions in pregnancy diseases (Pinto et al., 2023). The correlation analysis of differential microbiota with lipid metabolism and circulating SCFA metabolites suggested that the relative abundance of *Dorea* was negatively correlated with TCHO and LDL, while the relative abundance of *Dorea* and *Paraprevotella* was positively correlated with hexanoic acid. *Paraprevotella* is a butyrate-producing bacterium, and *Dorea* is an SCFA-producing bacterium (Graf et al., 2019). A previous study showed a negative correlation between the abundance of *Paraprevotella* and serum TG levels (Yang et al., 2023). GOS prebiotics, with breast milk-derived components, may promote the production of SCFAs such as hexanoic acid by targeting the gut microbiota, thereby improving lipid metabolism. Furthermore, PICRUSt2 is a reliable metabolic prediction tool and should be used further (Douglas et al., 2020). In future research, we will use PICRUSt2 to infer the abundance of key metabolic pathways, including KEGG and MetaCyc pathways, identify enzyme families, and predict functional traits encoded by the gut microbiota. By integrating these predicted functional profiles with our existing metabolomics and clinical data, we will be better positioned to build a more comprehensive understanding of microbiome-host interactions.

Several limitations of our study should be considered. Although we collected self-reported data on lifestyle, diet, and physical activity, the variability and subjective nature of this information precluded its use as a reliable covariate in our core analyses. Future investigations with prospectively designed and quantitatively rigorous assessments of these confounders are warranted to confirm our findings. Future research should aim to incorporate more precise and comprehensive measures of lifestyle and environmental exposures (Zhang et al., 2024). Specifically, the use of validated food-frequency questionnaires, accelerometry for physical activity, and detailed assessments of other lifestyle factors would be invaluable for controlling for potential confounding and elucidating the independent effects of the microbial and metabolic pathways identified in this study.

## 5 Conclusion

GOS preparations containing ingredients derived from human milk may target the gut microbiota to promote the production of hexanoic acid, thereby improving lipid metabolism and inflammation, and may be beneficial for overweight and obese people with GDM.

## Data Availability

The authors confirm that the data supporting the findings of this study are available within the article and its Supplementary materials. The datasets generated or analyzed during the current study are available in the NCBI database, SRA data (Accession number: PRJNA925813).

## References

[ref1] Babadi M. Khorshidi A. Aghadavood E. Samimi M. Kavossian E. Bahmani F. . (2019). The effects of probiotic supplementation on genetic and metabolic profiles in patients with gestational diabetes mellitus: a randomized, double-blind, placebo-controlled trial. Probiotics Antimicrob. Proteins 11, 1227–1235. doi: 10.1007/s12602-018-9490-z, 30535534

[ref2] Bamberger C. Rossmeier A. Lechner K. Wu L. Waldmann E. Fischer S. . (2018). A walnut-enriched diet affects gut microbiome in healthy Caucasian subjects: a randomized, controlled trial. Nutrients 10:244. doi: 10.3390/nu10020244, 29470389 PMC5852820

[ref3] Barnett D. J. M. Endika M. F. Klostermann C. E. Gu F. Thijs C. Nauta A. . (2023). Human milk oligosaccharides, antimicrobial drugs, and the gut microbiota of term neonates: observations from the KOALA birth cohort study. Gut Microbes 15:2164152. doi: 10.1080/19490976.2022.2164152, 36617628 PMC9833409

[ref4] Carlson J. L. Erickson J. M. Hess J. M. Gould T. J. Slavin J. L. (2017). Prebiotic dietary fiber and gut health: comparing the *in vitro* fermentations of beta-glucan, inulin and xylooligosaccharide. Nutrients 9:1361. doi: 10.3390/nu9121361, 29244718 PMC5748811

[ref5] Cheng W. Lu J. Lin W. Wei X. Li H. Zhao X. . (2018). Effects of a galacto-oligosaccharide-rich diet on fecal microbiota and metabolite profiles in mice. Food Funct. 9, 1612–1620. doi: 10.1039/c7fo01720k, 29465126

[ref6] Crusell M. K. W. Hansen T. H. Nielsen T. Allin K. H. Rühlemann M. C. Damm P. . (2018). Gestational diabetes is associated with change in the gut microbiota composition in third trimester of pregnancy and postpartum. Microbiome 6:89. doi: 10.1186/s40168-018-0472-x, 29764499 PMC5952429

[ref7] Douglas G. M. Maffei V. J. Zaneveld J. R. Yurgel S. N. Brown J. R. Taylor C. M. . (2020). PICRUSt2 for prediction of metagenome functions. Nat. Biotechnol. 38, 685–688. doi: 10.1038/s41587-020-0548-6, 32483366 PMC7365738

[ref8] Gibson G. R. Hutkins R. Sanders M. E. Prescott S. L. Reimer R. A. Salminen S. J. . (2017). Expert consensus document: the international scientific Association for Probiotics and Prebiotics (ISAPP) consensus statement on the definition and scope of prebiotics. Nat. Rev. Gastroenterol. Hepatol. 14, 491–502. doi: 10.1038/nrgastro.2017.75, 28611480

[ref9] Gomez-Arango L. F. Barrett H. L. Wilkinson S. A. Callaway L. K. McIntyre H. D. Morrison M. . (2018). Low dietary fiber intake increases Collinsella abundance in the gut microbiota of overweight and obese pregnant women. Gut Microbes 9, 189–201. doi: 10.1080/19490976.2017.1406584, 29144833 PMC6219589

[ref10] Graf D. Monk J. M. Lepp D. Wu W. McGillis L. Roberton K. . (2019). Cooked red lentils dose-dependently modulate the colonic microenvironment in healthy C57Bl/6 male mice. Nutrients 11:1853. doi: 10.3390/nu11081853, 31405019 PMC6724071

[ref11] Hasain Z. Mokhtar N. M. Kamaruddin N. A. Mohamed Ismail N. A. Razalli N. H. Gnanou J. V. . (2020). Gut microbiota and gestational diabetes mellitus: a review of host-gut microbiota interactions and their therapeutic potential. Front. Cell. Infect. Microbiol. 10:188. doi: 10.3389/fcimb.2020.00188, 32500037 PMC7243459

[ref12] Hiel S. Gianfrancesco M. A. Rodriguez J. Portheault D. Leyrolle Q. Bindels L. B. . (2020). Link between gut microbiota and health outcomes in inulin -treated obese patients: lessons from the Food4Gut multicenter randomized placebo-controlled trial. Clin. Nutr. 39, 3618–3628. doi: 10.1016/j.clnu.2020.04.005, 32340903

[ref13] Karamali M. Dadkhah F. Sadrkhanlou M. Jamilian M. Ahmadi S. Tajabadi-Ebrahimi M. . (2016). Effects of probiotic supplementation on glycaemic control and lipid profiles in gestational diabetes: a randomized, double-blind, placebo-controlled trial. Diabetes Metab. 42, 234–241. doi: 10.1016/j.diabet.2016.04.009, 27209439

[ref14] Kijmanawat A. Panburana P. Reutrakul S. Tangshewinsirikul C. (2019). Effects of probiotic supplements on insulin resistance in gestational diabetes mellitus: a double-blind randomized controlled trial. J. Diabetes Investig. 10, 163–170. doi: 10.1111/jdi.12863, 29781243 PMC6319478

[ref15] Kim M. H. Kang S. G. Park J. H. Yanagisawa M. Kim C. H. (2013). Short-chain fatty acids activate GPR41 and GPR43 on intestinal epithelial cells to promote inflammatory responses in mice. Gastroenterology 145, 396–406.e10. doi: 10.1053/j.gastro.2013.04.056, 23665276

[ref16] Koren O. Goodrich J. K. Cullender T. C. Spor A. Laitinen K. Bäckhed H. K. . (2012). Host remodeling of the gut microbiome and metabolic changes during pregnancy. Cell 150, 470–480. doi: 10.1016/j.cell.2012.07.008 22863002 PMC3505857

[ref17] La Fata G. Weber P. Mohajeri M. H. (2018). Probiotics and the gut immune system: indirect regulation. Probiotics Antimicrob. Proteins 10, 11–21. doi: 10.1007/s12602-017-9322-6, 28861741 PMC5801397

[ref18] Marco M. L. Sanders M. E. Gänzle M. Arrieta M. C. Cotter P. D. De Vuyst L. . (2021). The international scientific Association for Probiotics and Prebiotics (ISAPP) consensus statement on fermented foods. Nat. Rev. Gastroenterol. Hepatol. 18, 196–208. doi: 10.1038/s41575-020-00390-5, 33398112 PMC7925329

[ref19] Metzger B. E. Gabbe S. G. Persson B. Buchanan T. A. Catalano P. A. Damm P. . (2010). International association of diabetes and pregnancy study groups recommendations on the diagnosis and classification of hyperglycemia in pregnancy. Diabetes Care 33, 676–682. doi: 10.2337/dc09-1848 20190296 PMC2827530

[ref20] Miao M. Wang Q. Wang X. Fan C. Luan T. Yan L. . (2022). The protective effects of inulin-type Fructans against high-fat/sucrose diet-induced gestational diabetes mice in association with gut microbiota regulation. Front. Microbiol. 13:832151. doi: 10.3389/fmicb.2022.832151, 35495651 PMC9048744

[ref21] Mokkala K. Paulin N. Houttu N. Koivuniemi E. Pellonperä O. Khan S. . (2021). Metagenomics analysis of gut microbiota in response to diet intervention and gestational diabetes in overweight and obese women: a randomised, double-blind, placebo-controlled clinical trial. Gut 70, 309–318. doi: 10.1136/gutjnl-2020-321643, 32839200

[ref22] Müller M. Hernández M. A. G. Goossens G. H. Reijnders D. Holst J. J. Jocken J. W. E. . (2019). Circulating but not faecal short-chain fatty acids are related to insulin sensitivity, lipolysis and GLP-1 concentrations in humans. Sci. Rep. 9:12515. doi: 10.1038/s41598-019-48775-0, 31467327 PMC6715624

[ref23] Perdijk O. van Baarlen P. Fernandez-Gutierrez M. M. van den Brink E. Schuren F. H. J. Brugman S. . (2019). Sialyllactose and Galactooligosaccharides promote epithelial barrier functioning and distinctly modulate microbiota composition and short chain fatty acid production in vitro. Front. Immunol. 10:94. doi: 10.3389/fimmu.2019.00094, 30809221 PMC6380229

[ref24] Pinto Y. Frishman S. Turjeman S. Eshel A. Nuriel-Ohayon M. Shrossel O. . (2023). Gestational diabetes is driven by microbiota-induced inflammation months before diagnosis. Gut 72, 918–928. doi: 10.1136/gutjnl-2022-328406 36627187 PMC10086485

[ref25] Priyadarshini M. Thomas A. Reisetter A. C. Scholtens D. M. Wolever T. M. S. Josefson J. L. . (2014). Maternal short-chain fatty acids are associated with metabolic parameters in mothers and newborns. Transl. Res. 164, 153–157. doi: 10.1016/j.trsl.2014.01.012, 24530607 PMC4156825

[ref26] Ríos-Covián D. Ruas-Madiedo P. Margolles A. Gueimonde M. de Los Reyes-Gavilán C. G. Salazar N. (2016). Intestinal short chain fatty acids and their link with diet and human health. Front. Microbiol. 7:185. doi: 10.3389/fmicb.2016.00185, 26925050 PMC4756104

[ref27] Salazar N. Dewulf E. M. Neyrinck A. M. Bindels L. B. Cani P. D. Mahillon J. . (2014). Inulin-type fructans modulate intestinal *Bifidobacterium* species populations and decrease fecal short-chain fatty acids in obese women. Clin. Nutr. 34, 501–507. doi: 10.1016/j.clnu.2014.06.001 24969566

[ref28] Sun Z. Pan X.-F. Li X. Jiang L. Hu P. Wang Y. . (2023). The gut microbiome dynamically associates with host glucose metabolism throughout pregnancy: longitudinal findings from a matched case-control study of gestational diabetes mellitus. Adv. Sci. 10:e2205289. doi: 10.1002/advs.202205289, 36683149 PMC10074094

[ref29] Tang Z. Shao T. Gao L. Yuan P. Ren Z. Tian L. . (2023). Structural elucidation and hypoglycemic effect of an inulin-type fructan extracted from *Stevia rebaudiana* roots. Food Funct. 14, 2518–2529. doi: 10.1039/d2fo03687h, 36820831

[ref30] van der Beek C. M. Bloemen J. G. van den Broek M. A. Lenaerts K. Venema K. Buurman W. A. . (2015). Hepatic uptake of rectally administered butyrate prevents an increase in systemic butyrate concentrations in humans. J. Nutr. 145, 2019–2024. doi: 10.3945/jn.115.211193, 26156796

[ref31] Vulevic J. Juric A. Walton G. E. Claus S. P. Tzortzis G. Toward R. E. . (2015). Influence of galacto-oligosaccharide mixture (B-GOS) on gut microbiota, immune parameters and metabonomics in elderly persons. Br. J. Nutr. 114, 586–595. doi: 10.1017/s0007114515001889, 26218845

[ref32] Wan J. An L. Ren Z. Wang S. Yang H. Ma J. (2023). Effects of galactooligosaccharides on maternal gut microbiota, glucose metabolism, lipid metabolism and inflammation in pregnancy: a randomized controlled pilot study. Front. Endocrinol. 14:1034266. doi: 10.3389/fendo.2023.1034266, 36777355 PMC9911812

[ref33] Wang K. Duan F. Sun T. Zhang Y. Lu L. (2023). Galactooligosaccharides: synthesis, metabolism, bioactivities and food applications. Crit. Rev. Food Sci. Nutr. 64, 6160–6176. doi: 10.1080/10408398.2022.2164244, 36632761

[ref34] Wang X. Liu H. Li Y. Huang S. Zhang L. Cao C. . (2020). Altered gut bacterial and metabolic signatures and their interaction in gestational diabetes mellitus. Gut Microbes 12, 1840765–1840713. doi: 10.1080/19490976.2020.1840765, 33222612 PMC7714515

[ref35] Wang S. Liu Y. Qin S. Yang H. (2022). Composition of maternal circulating short-chain fatty acids in gestational diabetes mellitus and their associations with placental metabolism. Nutrients 14:3727. doi: 10.3390/nu14183727, 36145103 PMC9505713

[ref36] Weitkunat K. Schumann S. Nickel D. Kappo K. A. Petzke K. J. Kipp A. P. . (2016). Importance of propionate for the repression of hepatic lipogenesis and improvement of insulin sensitivity in high-fat diet-induced obesity. Mol. Nutr. Food Res. 60, 2611–2621. doi: 10.1002/mnfr.201600305, 27467905 PMC5215627

[ref37] Wu Y. Zhang X. Pi Y. Han D. Feng C. Zhao J. . (2021). Maternal galactooligosaccharides supplementation programmed immune defense, microbial colonization and intestinal development in piglets. Food Funct. 12, 7260–7270. doi: 10.1039/d1fo00084e, 34165467

[ref38] Yang X. Zhang M. Zhang Y. Wei H. Guan Q. Dong C. . (2023). Ecological change of the gut microbiota during pregnancy and progression to dyslipidemia. NPJ Biofilms Microbiomes 9:14. doi: 10.1038/s41522-023-00383-7, 37012285 PMC10070613

[ref39] Zhang L. Wang F. Tashiro S. Liu P. J. (2024). Effects of dietary approaches and exercise interventions on gestational diabetes mellitus: a systematic review and Bayesian network meta-analysis. Adv. Nutr. 15:100330. doi: 10.1016/j.advnut.2024.100330, 39481539 PMC11629230

[ref40] Zhang S. Wang H. Zhu M.-J. (2019). A sensitive GC/MS detection method for analyzing microbial metabolites short chain fatty acids in fecal and serum samples. Talanta 196, 249–254. doi: 10.1016/j.talanta.2018.12.049, 30683360

[ref41] Zhao L. Zhang F. Ding X. Wu G. Lam Y. Y. Wang X. . (2018). Gut bacteria selectively promoted by dietary fibers alleviate type 2 diabetes. Science 359, 1151–1156. doi: 10.1126/science.aao5774, 29590046

