# Supplementary material for: Effects of galactooligosaccharides on maternal metabolism and the gut microbiota during pregnancy
Source: Front Microbiol. 2026 Jan 6;16:1679308. doi: 10.3389/fmicb.2025.1679308 (PMC12815777; doi:10.3389/fmicb.2025.1679308)
Supplement: Supplementary file 1 [file Data_Sheet_1.docx]

**Supplementary material**

Table S1 Chromatographic gradient program

| Time (min) | Aqueous phase (A, C %) | Organic phase (B, D %) |
| --- | --- | --- |
| 0.0-0.5 | 98 | 2 |
| 0.5-6 | 98-50 | 2-50 |
| 6-10 | 50-2 | 50-98 |
| 10-14 | 2-98 | 98-2 |
| 16-21 | 98 | 2 |

Table S2 Mass spectrometry parameters

| Parameter | Setting |
| --- | --- |
| **Scan mode** | Positive and negative ions scanned separately |
| **Ionization source** | ESI (Electrospray ionization) |
| **Electrospray voltage** | 2.5 kV (Positive); 2.5 kV (Negative) |
| **Capillary temperature** | 325 °C |
| **Sheath gas flow rate** | 50 Arb |
| **Aux gas heater temperature** | 300 °C |

Table S3 List of specific results of metabolic pathways

| Pathway Name | Total Cmpd | Hits | Raw p | -Log(p) | FDR | Impact |
| --- | --- | --- | --- | --- | --- | --- |
| **Butanoate metabolism** | **40** | **3** | **0.018211** | **4.0058** | **0.21415** | **0.1077** |
| Synthesis and degradation of ketone bodies | 6 | 1 | 0.025736 | 3.6599 | 0.21415 | 0 |
| **Propanoate metabolism** | **35** | **2** | **0.029363** | **3.528** | **0.21415** | **0.03027** |
| **Cysteine and methionine metabolism** | **56** | **3** | **0.039907** | **3.2212** | **0.21415** | **0.11707** |
| Histidine metabolism | 44 | 1 | 0.041171 | 3.19 | 0.21415 | 0.00861 |
| Vitamin B6 metabolism | 32 | 2 | 0.058839 | 2.833 | 0.21415 | 0.07958 |
| Pantothenate and CoA biosynthesis | 27 | 2 | 0.062677 | 2.7698 | 0.21415 | 0.18014 |
| Ascorbate and aldarate metabolism | 45 | 3 | 0.062872 | 2.7667 | 0.21415 | 0.13849 |
| Pyruvate metabolism | 32 | 1 | 0.065086 | 2.732 | 0.21415 | 0.18254 |
| Glycolysis or Gluconeogenesis | 31 | 1 | 0.065086 | 2.732 | 0.21415 | 0.0953 |
| Taurine and hypotaurine metabolism | 20 | 1 | 0.065086 | 2.732 | 0.21415 | 0.02158 |
| Pentose and glucuronate interconversions | 53 | 1 | 0.065086 | 2.732 | 0.21415 | 0 |
| Terpenoid backbone biosynthesis | 33 | 1 | 0.065086 | 2.732 | 0.21415 | 0 |
| Nicotinate and nicotinamide metabolism | 44 | 2 | 0.065101 | 2.7318 | 0.21415 | 0.0015 |
| Pentose phosphate pathway | 32 | 2 | 0.065557 | 2.7248 | 0.21415 | 0.09801 |


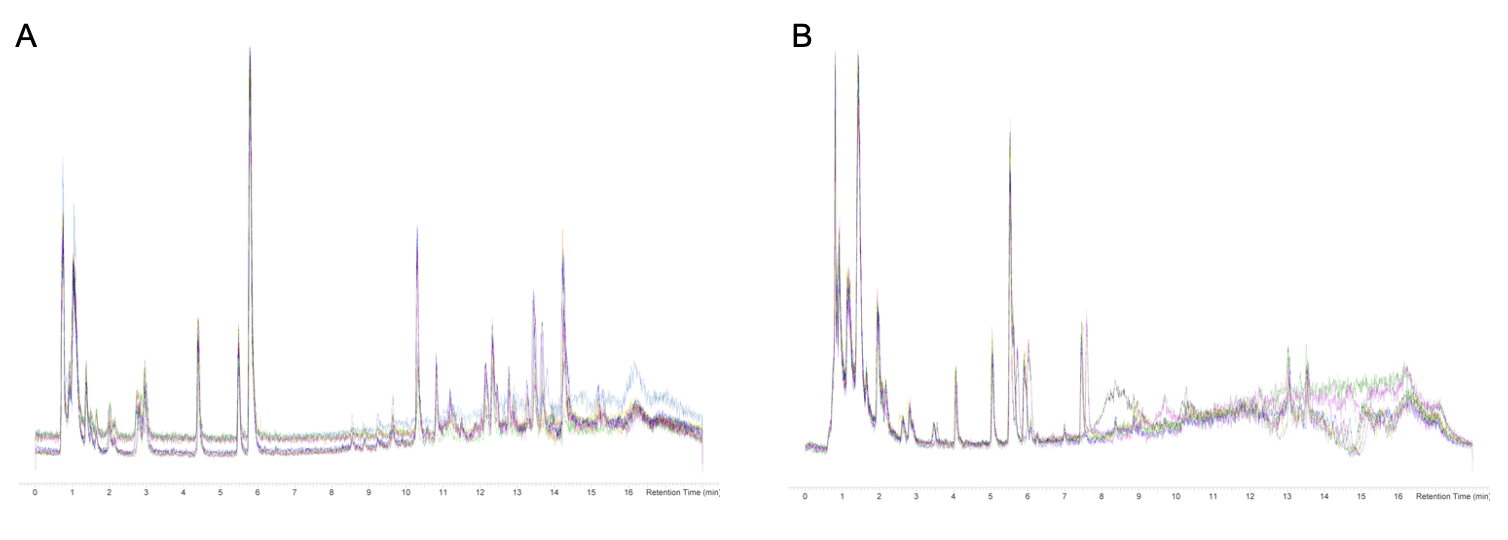


Figure S1. Total ion chromatograms of QC. (A) Total ion chromatograms of QC in positive ion mode. (B) Total ion chromatograms of QC in negative ion mode.


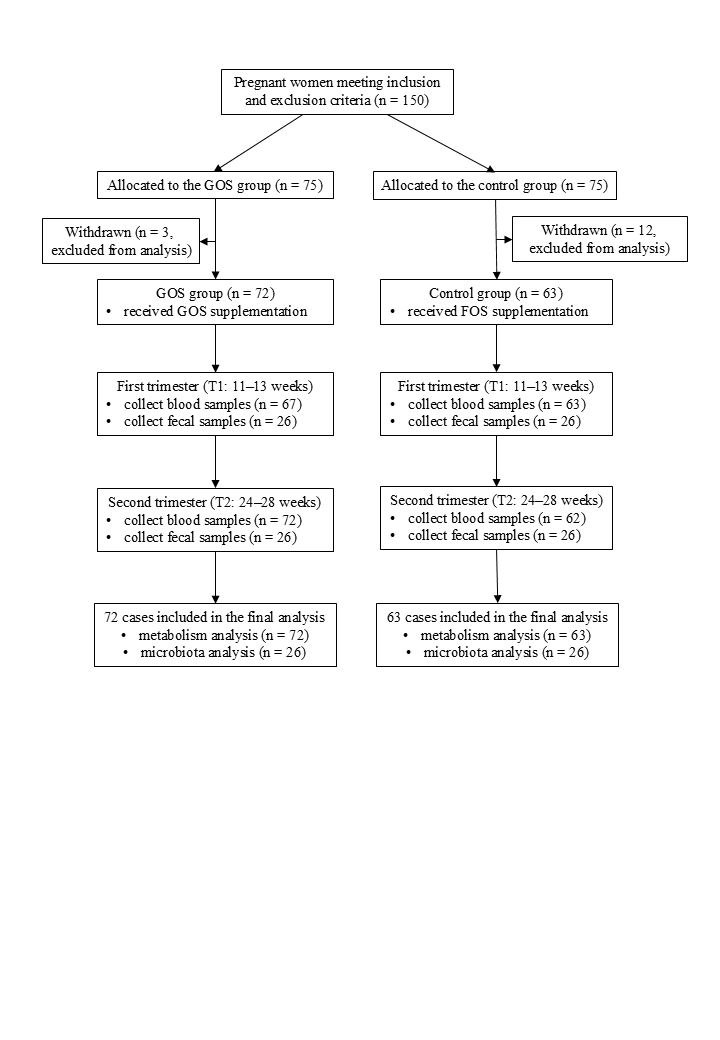


Figure S2 Flow chart of participants and analyses through the study.


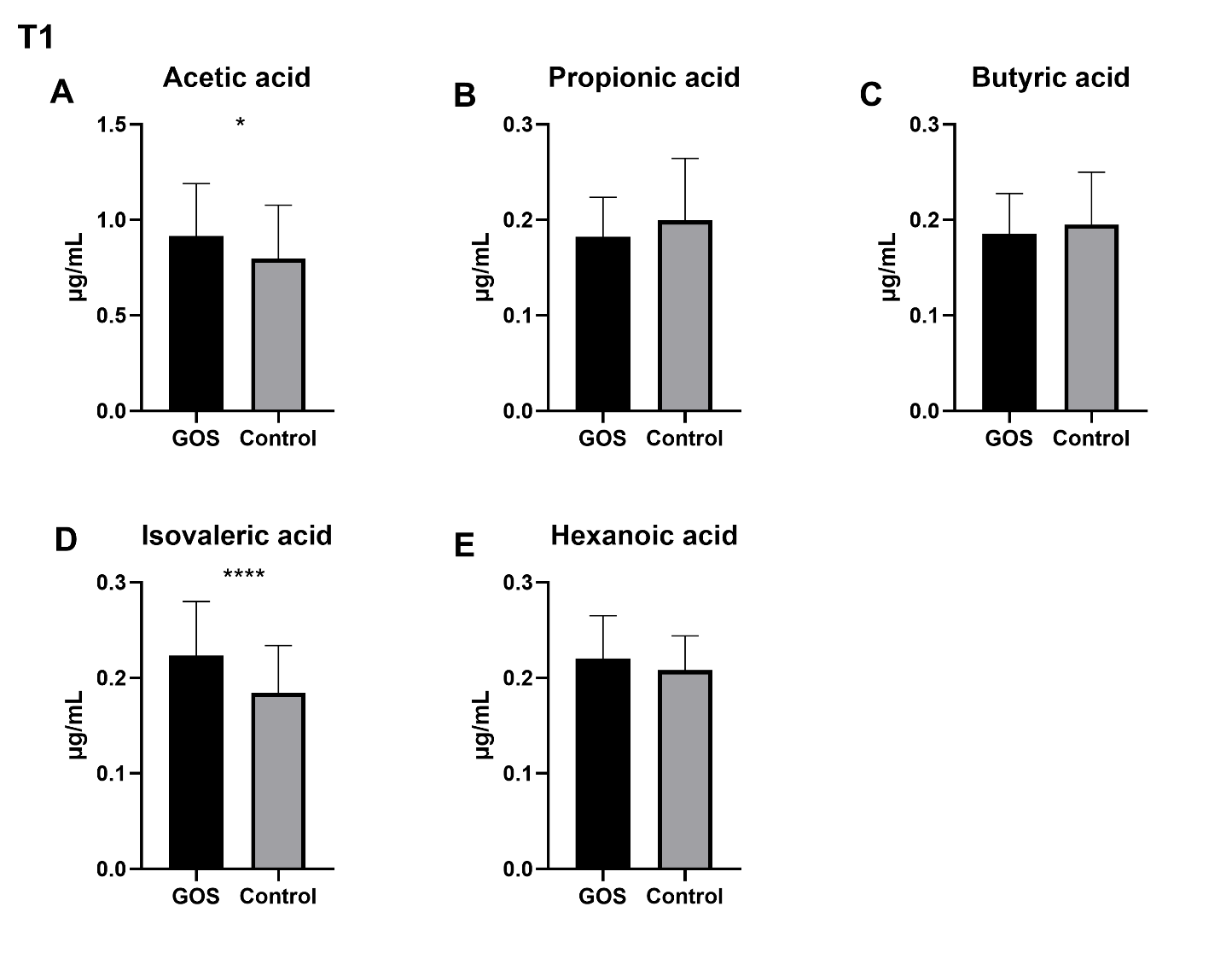
Figure S3 Circulating SCFAs content levels in T1. (A-E) The circulating levels of acetic acid, propionic acid, butyric acid, isovaleric acid, and hexanoic acid of the GOS and control groups in T1.


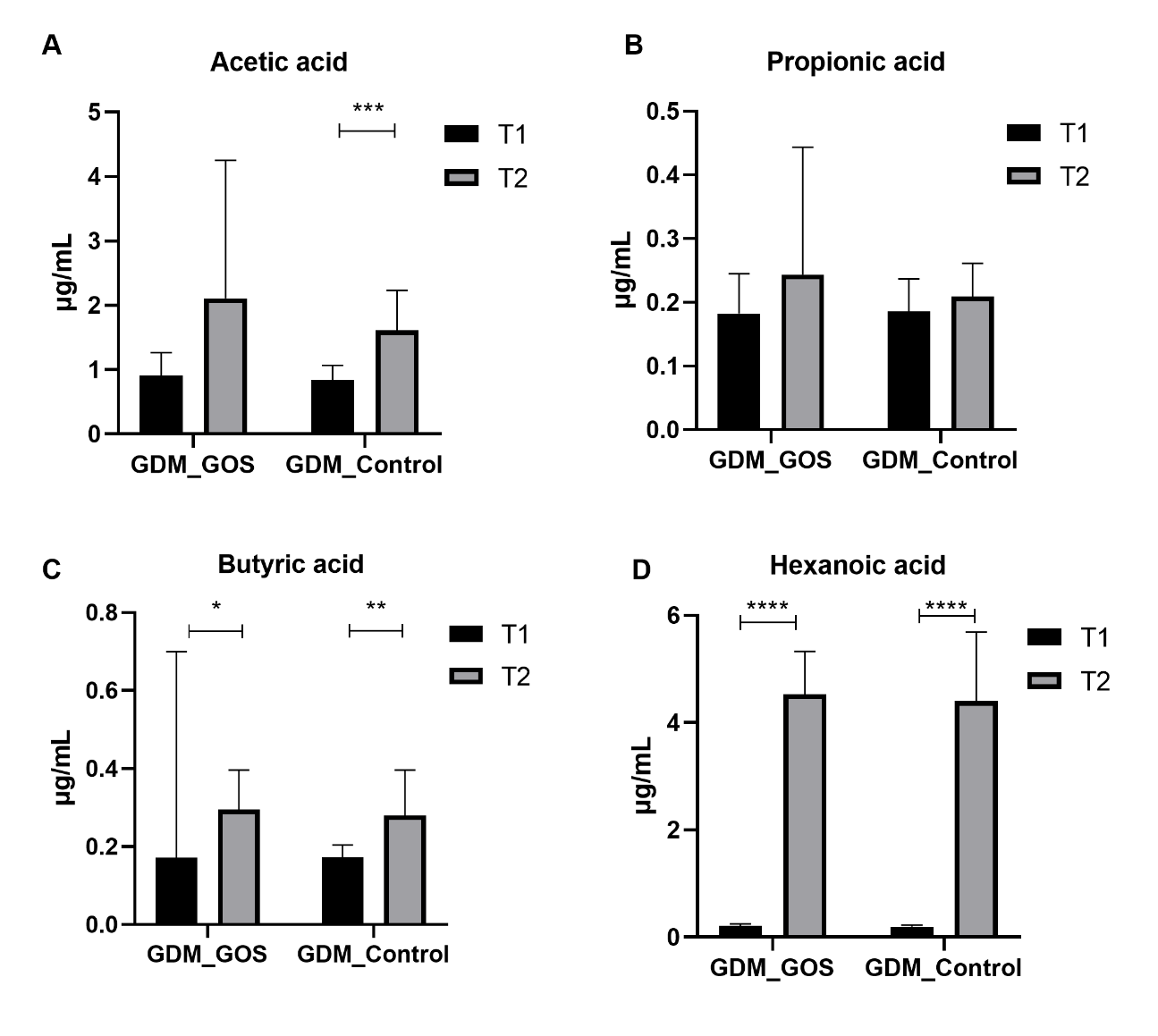


Figure S4 SCFAs levels in overweight and obese GDM pregnant women. (A-D) The circulating levels of acetic acid, propionic acid, butyric acid, and hexanoic acid in in either group in T1and T2.


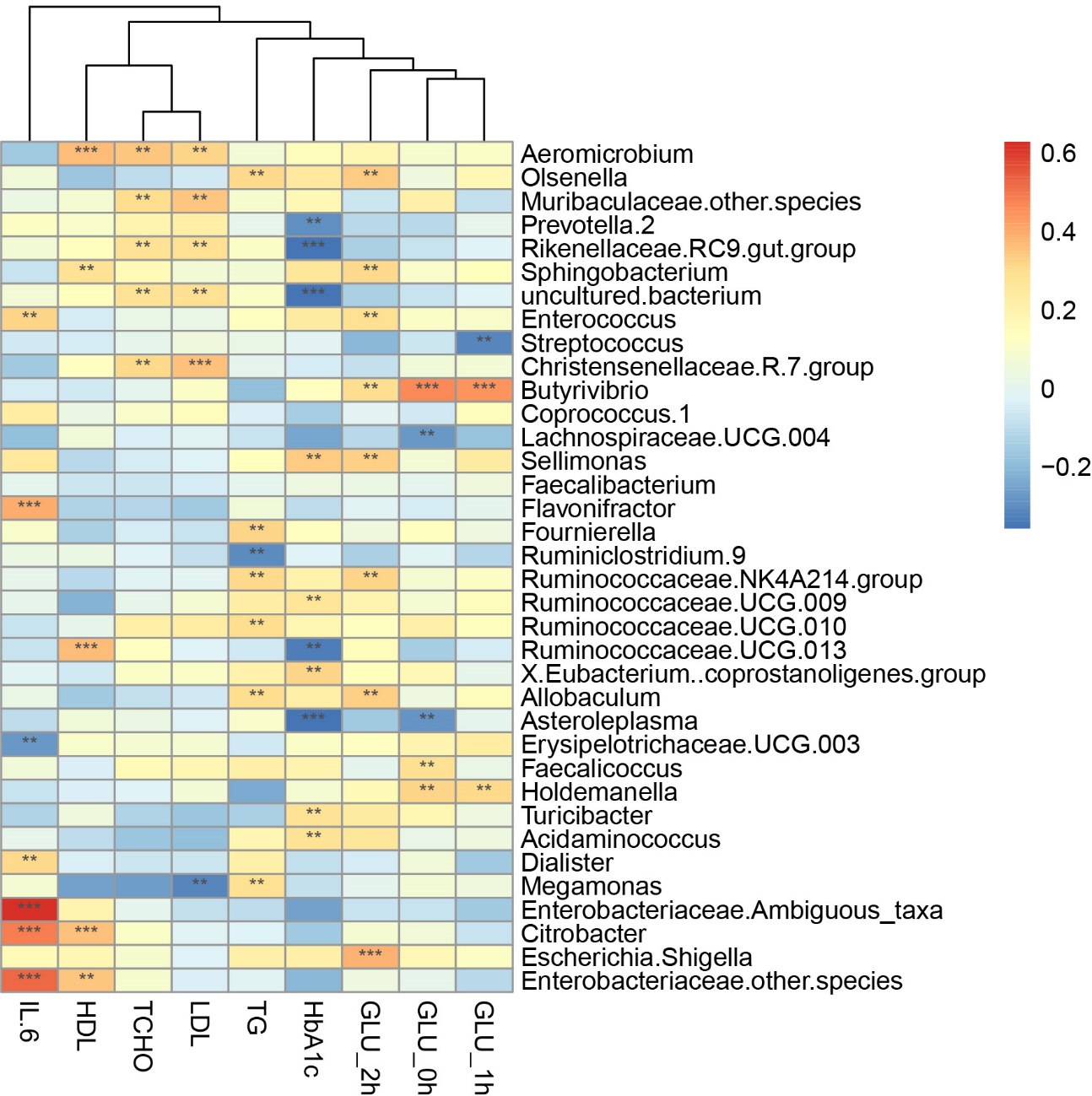


Figure S5 Correlation of relative abundance of bacteria at genus level with glycolipid metabolism and inflammatory factors. The correlation heatmap between the relative abundance of bacteria at genus level and LDL, TCHO, HDL, TG, GLU 0h, GLU 1h, GLU 2h, HbA1c, and IL-6. Red squares and blue squares indicate positive and negative associations, respectively. ***P < 0.01, **P < 0.05, *P < 0.1.


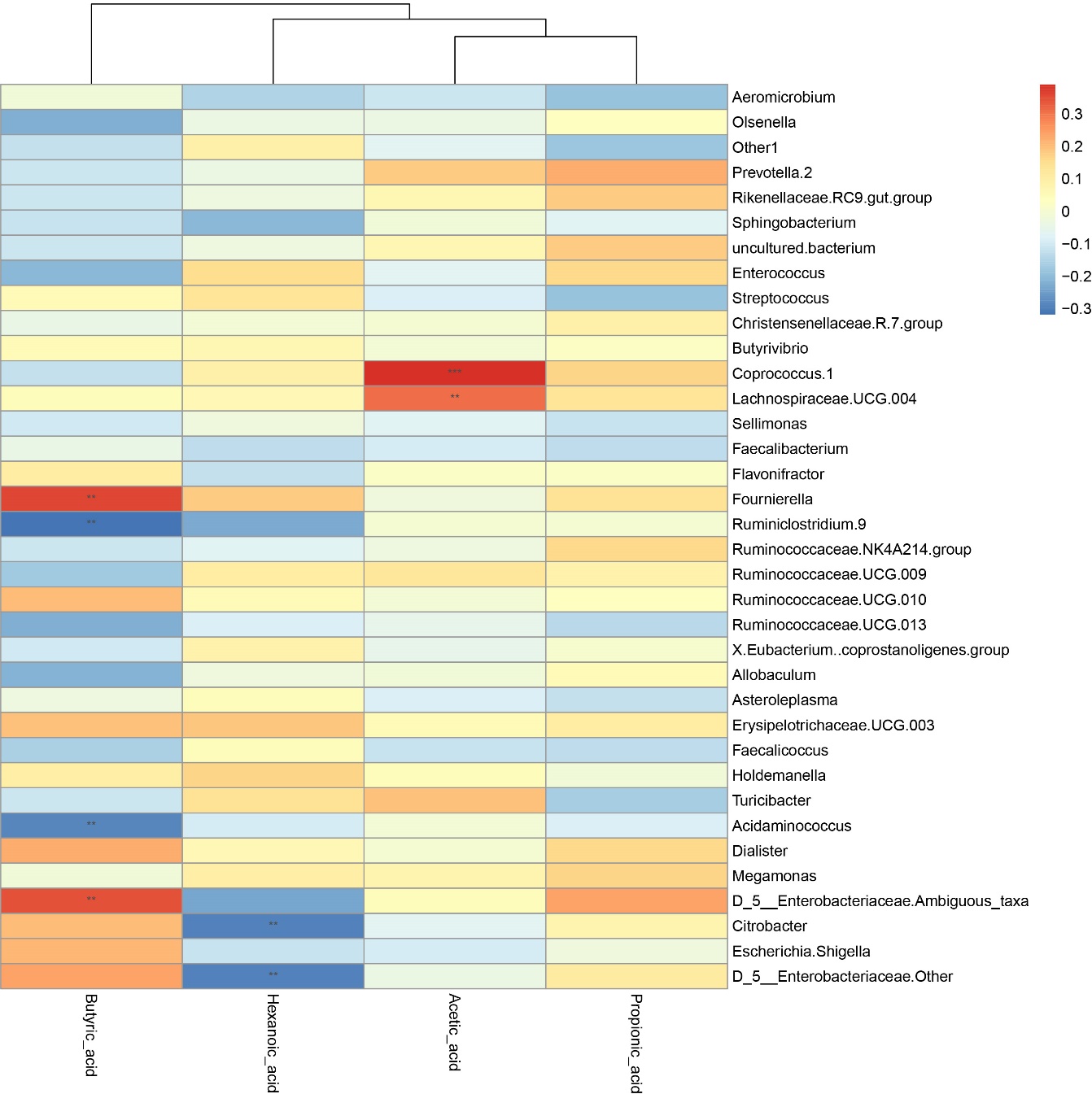


Figure S6 Correlation between the relative abundance of bacteria at genus level and SCFAs. The correlation heatmap between the relative abundance of bacteria at genus level and acetic acid, propionic acid, butyric acid, and hexanoic acid. Red squares and blue squares indicate positive and negative associations, respectively. ***P < 0.01, **P < 0.05, *P < 0.1.
